# New Inventions

**Published:** 1913-04

**Authors:** 


### NEW INVENTIONS

Geo. H. Houghton, Albany, N. Y., Surgical Apparatus, No. 1,055,462.

Richard Blunk, Wesel, Germany, Surgical Tweezers, No. 1,053,149.

Edw. Bohlander, Pekin, Ill., Ankle-Joint for Artificial Limbs, No. 1,053,352.

C. W. Sillin, Stuttgart, Ark., Abdominospinal Support or Splint, No. 1,052,523.

Albert E. Stoll, Zollikon, Switzerland, Apparatus for Making Hydrogen Peroxide, No. 1,052,626.

E. F. Petersen, Frankfort-on-the-Main, Germany, Pessary, No. 1,052,687.

Wm. W. Evans, Hamilton, Va., Dental Instrument, No. 1,052,806.

Chas. J. Greenstreet, Indianapolis, Ind., Process of Making Nitrogenous Compounds, No. 1,052,815.

A. J. Curlee, Waxahachie, Tex., Impregnator, No. 1,052,-895.

Geo. Zitzmann, West Hoboken, N. J., and Frank Shoreys, New York, N. Y., Bottle Label, No. 1,052,991.

Mattie E. Siebert, Spokane, Wash., Catamenial Sack, No. 1,053,069.

Albert Wolff, Cologne, Germany, Process for Preparation of Formates of Chromium Alluminium and Iron, No. 1,054,735.

---

Harry Spirs, San Francisco, Cal., Instruments used for Aiagnostic Purposes in Obsterical Practice, No. 1,054,802.

---

Charles L. Allen, Salt Lake City, Utah, and Samuel C. Miles, Price, Utah, Hair Drying Apparatus, No. 1,054,813.

---

F. J. Suchanek, New York, N. Y., Hydrothermometer, No. 1,054,879.

---

Q. E. Packard, Montreal, Quebec, Canada, Bunion Protector. No. 1,054,934.

---

William B. Wilcox, Ypsilanti, Mich., Crutch Tip, No. 1,055,-111.

---

Charles F. Hyde, Chicago, Ill., Medicine Dropper. No. 1,055,-180.

---

Julius I. Klepper, New York, N. Y., Syringe, No. 1,055,188.

---

Benjamin Seacombe, Pendleton, near Manchester, England, assignor to Thompson & Capper, Manchester, England, Magneto-Electric Massaging Machine, No. 1,055,236.

---

Jesse G. Grantham, Jacksonville, Ill., Poison Indicating Bottle, No. 1,055,268.

